# Supervised and unsupervised rehabilitation of visual field defect: cohort investigation of eye movement training at a clinical setting and at home

**DOI:** 10.1007/s00221-025-07105-9

**Published:** 2025-06-09

**Authors:** Valentina Varalta, Sigrid Kenkel, Samuel Johnson, Cristina Fonte, Nicola Smania, Arash Sahraie

**Affiliations:** 1 Neuromotor and Cognitive Rehabilitation Research Centre, Department of Neuroscience, Biomedicine and Movement Sciences, University of Verona, Verona, Italy; 2 Neurorehabilitation Unit, AOUI Verona, Verona, Italy; 3 Authur has been added posthumously, NovaVision GmbH, Magdeburg, Germany; 4 School of Psychology, University of Aberdeen, William Guild Building, Aberdeen, AB24 3FX UK

**Keywords:** Hemianopia, Eye movement, Vision, Rehabilitation, Partial blindness

## Abstract

Lesions along the visual pathways can lead to areas of blindness, which can extend to an entire hemifield (hemianopia). Hemianopic patients often have abnormal eye-movements which hampers their interaction with their immediate surrounds, adversely affecting their quality of life. Compensatory rehabilitation techniques are aimed at improving eye-movement efficacy enabling patients to make better use of their sighted field to compensate for the sight loss. NeuroEyeCoach (NEC) is a compensatory vision therapy that can be accessed at home and is effective in improving visual search performance and reducing perceived disability. We have compared objective and subjective assessments of visual function before and after vision rehabilitation in a cohort of patients that accessed NEC at home (*N* = 95) and a group of patients that completed the training under clinical supervision in a rehabilitation clinic (*N* = 31). Use of NEC led to improvements in both objective measures of visual function such as reduced visual search times, lower search errors, and faster completion of a cancellation task as well as reduced subjective reports of disability. The objective measures showed a larger improvement in those undergoing rehabilitation in the clinic settings compared to the home cohort, nevertheless, there was no cohort x training interaction for perceived improvements in subjective reports of disability. This indicates no significant differences on the effect of training on activities of daily living between the groups. The findings demonstrate that compensatory eye movement training is an effective tool for rehabilitation of vision loss when used in clinical settings or accessed remotely from home.

## Introduction

Stroke is the major cause of brain injury, and in chronic cases, depending on the site and extent of the injury, may leave the patient with significant and lasting sensory, motor, or cognitive impairments. As a significant number of brain areas are either exclusively or partially involved in processing of visual information, a significant proportion of cases (65%) (Hepworth et al. 2016) suffer from some impairment of visual processing (Rowe et al. 2008, 2019). The label of visual impairment covers a wide range of symptoms and dysfunctions, including blurred vision, reduced contrast sensitivity, eye movement disorders, reduced colour or form vision and visual field loss to name a few. Despite these significant visual deficits, there are some 40% of stroke survivors who do not or cannot report visual losses (Hepworth et al. 2021). These deficits may occur on their own or in combination with others. Visual field loss has a high incidence (50%) (Hepworth et al. 2016; Zhang et al. 2006) and in cases of post chiasmatic brain injury the loss covers the same part of the visual field in both eyes, hence the resultant blindness is referred to as homonymous hemianopia. Characteristically, stroke is a sudden life event and unlike the progressive degenerative diseases where patients can develop adaptive strategies over lengthy periods of time (Sand et al. 2013), the wide range of deficits and resultant disabilities are spontaneous. Although some recovery of function may take place in the acute stage post-stroke (0–6 months), at the chronic stage the deficits are thought to be permanent without active interventions (Ali et al. 2013; Nys et al. 2005; Zhang et al. 2006).

The therapeutic management of visual field loss can be subdivided into three approaches, namely substitutions, restoration, and compensatory techniques. A substitution technique involves using prisms embedded in spectacles and the field of view at direct gaze can be extended into the blind areas by a few degrees allowing improved obstacle detection, aiding patient’s navigation skills (Bowers et al. 2014). This technique is not widely used as, in long term usage, the tolerance for manipulation of optical properties is low and their side effects include double vision (Rowe et al. 2017). Also benefits are restricted to the times that the device is worn (Bowers et al. 2014). Restoration techniques are aimed at reducing the extent of blindness by increasing visual sensitivity within the impaired field. These techniques make use of repeated stimulation coupled with perceptual learning (Pollock et al. 2019). Systematic repeated stimulation using flashing gratings (NeuroEyeTherapy, NeET) (Sahraie et al. 2010; Trevethan et al. 2012), Vision Restoration Therapy (VRT) (Kasten et al. 1999), and stimulation using moving dots (Das et al. 2014; Huxlin et al. 2009) are three examples of techniques that rely on repeated stimulation of specific locations within the field defect, and over extended periods of time can lead to some recovery of visual fields. There is now a significant body of research reporting the behavioural benefits of restoration techniques (Bouwmeester et al. 2007; Glisson 2006) and the associated changes in brain activity and function (Ajina et al. 2021; Jobke et al. 2009; Marshall et al. 2008; Willis et al. 2025). There remain two main challenges for restoration techniques. Firstly, there is a wide range of individual differences on the extent of reported recovery with anything between none to substantial recovery of the visual field being reported (Bouwmeester et al. 2007; Glisson 2006; Leitner et al. 2023). As the location, size and extent of injury in each case is unique, the extent of recovery for an individual cannot yet be predicted (Sahraie et al. 2013). Secondly restoration interventions often require training over an extended period of time (3–6 months) (Sahraie 2007; Saionz et al. 2021). The combination of demands associated with lengthy therapy and unpredictability of the extent of recovery may not appeal to many of those affected by sight loss.

Compensatory therapies are aimed at improving detection of items in the blind field by enhancing eye movement scanning. The sudden onset of hemianopia after brain injury, affect the dynamics of eye movements for target detection in both sighted and blind fields in the acute phase (Zihl 1995; Zihl and Hebel 1997). The deficits persist in 60% of cases at the chronic phase (Zihl 1995). These deficits include shorter saccade amplitudes leading to a larger number of fixations, hence slowing exploratory eye movements (Meienberg et al. 1981; Pambakian 2000). Other behavioural characteristics include more emphasis on exploring objects within the sighted field rather than shifting gaze into the blind areas (Zihl 2010). Of note is that such behaviours are seen and persist when the hemianopic field damage is simulated in healthy observers under laboratory (Nowakowska et al. 2016; Schuett et al. 2009), indicating that the inefficient eye movement strategies are not caused as a direct result of damage to the neuronal mechanisms for eye movement control, but are likely to be as a result of changes in cognitive functions. Therefore, appropriate training is required to modify the global eye movement behaviour. Considerable research has shown that training patients on visual search tasks can lead to better detection and more efficient eye movements (Nowakowska et al. 2019). Indeed, compensatory techniques aimed at improving eye movements in hemianopic patients were recommended in systematic reviews (Ali et al. 2013; Pollock et al. 2019). Despite this promising backdrop, there is limited opportunity for those who can benefit from visual rehabilitation to access them. This is largely due to an overall lack of translation of technologies from basic science into clinical care. The detailed regulatory process for such translations takes significant time and resource as the software-based interventions are also classed within the medical device category in both the EU and the US.

NeuroEyeCoach (NEC) is an eye movement based compensatory approach to rehabilitation of visual field deficits following brain injury (Sahraie et al. 2016). NEC is a Class I CE marked medical device in the EU and is registered as an FDA 510(K) exempt medical device in the US. It consists of a series of visual search exercises and the software is adaptive to patient’s deficit. The in-built algorithms adjust the task difficulty based on the patient’s performance in order to improve search efficiency (Sahraie et al. 2016). As NEC is cloud-based, it can be accessed from home or clinical settings. A recent report on 296 patients completing the therapy online showed significant improvements in objective measures of performance in over 80% of cases (Sahraie et al. 2020). Importantly, self-reported measures of disability also improved in 66% of cases and these improvements were independent of patient’s age at brain injury or commencing the therapy, sex, lesion side, and time between injury and therapy.

The rehabilitation provisions and methods of delivery are variable across the countries in Europe and even various states in the US. They range from specialist care provided in clinical settings, homebased visits, or advice. There has been a significant drive to enhance access to digital technologies at home settings in Germany and the UK, nevertheless clinic-based models are more frequent across the EU and remotely accessed therapies are promoted in the US. The variability across the countries is largely driven by methods and mechanisms for cost recovery. NEC has been aimed at low-cost delivery ($300) to allow wide access. Anecdotally, one would expect that provision of therapies in clinical settings to lead to better engagement and compliance by patients, even if it merely reflects the mindset of preferring human interactions. It is therefore important to compare the objective and subjective measures of performance after vision rehabilitation in home-based patients and those accessing it in the clinical settings. The findings can potentially be useful in deciding modes of service delivery, such as care in the community or clinical settings. Here we report on these comparisons in a group of 95 patients that were home-based and 31 patients that attended daily rehabilitation sessions in a clinical setting.

## Methods

### Participants

We compared the performance of two patient groups undertaking compensatory visual rehabilitation following brain injury, using NeuroEyeCoach. The home-based group consisted of 95 consecutive patients that accessed and completed the therapy online. The clinic group were 31 consecutive patients that had completed the therapy whilst attending Neurorehabilitation unit of the Neuromotor and Cognitive Rehabilitation Research Centre, University of Verona. For the clinical group, visual field losses were verified by medical records. Patients in home-based group were either recommended to contact Nova Vision Inc. (www.novavision.com) by a medical practitioner or were self-referred after experiencing visual field loss post stroke. Similar to other compensatory vision therapies designed to be directly accessible to the patient population (Ong et al. 2015www.eyesearch.ucl.ac.uk), the self-reported visual field defects were not verified prior to access to the therapy. We have accessed the database for consecutive patients who had completed datasets and had not previously reported, up to the end of October 2020. Both therapy and data for clinic-based patients were stored on a standalone PC in the clinical settings and anonymised data for completed patients was downloaded. Ethical approval for the study was granted by the Psychology Ethics Committee, University of Aberdeen (PEC.4684.20212). All statistical analyses reported here are conducted using JASP 0.18.3. Bonferroni correction has been applied to all post-hoc p values reported. The data is accessible on Open Science Forum (osf.io/mpr4s).

Home-based group consisted of 69 male and 26 female, mean age at brain injury 53.74 years (SD = 18.25); with mean time between injury and start of therapy of 19.0 months (SD = 38.0). A total of 44 patients reported have left sided blindness, 47 right sided blindness and 4 patients reported that blindness had affected both sides. Clinic-based group consisted of 21 male and 10 female patients, mean age at brain injury 59.84 years (SD = 15.53); with mean injury-therapy time gap of 5.10 months (SD = 9.75). A total of 16 patients reported to have left sided blindness and 15 right sided blindness.

### Intervention

A full description of NEC has been previously outlined elsewhere (Sahraie et al. 2016, 2020). In brief, the software assesses the specification of IT equipment used by the patient to access NEC and appropriate recommendations on eye/head position and viewing distance are provided to the patient to ensure exposure to a visual field of ± 20°. Having provided the appropriate advice on set up and viewing distance to home group, the actual set up was not intended to be verifiable. The target size is also algorithmically adjusted to ensure those with low near visual acuity are able to detect and discriminate individual items on the screen. In a visual search task, the patient is required to report the target’s presence when target is detected or report the absence of a target item amongst distractors by pressing left or right mouse buttons respectively. In general, there are three ways to manipulate the task difficulty in order to improve search strategy. These are target/distractor visual similarity, set size (number of distractor items on the screen) and response time window. All the three techniques are used to systematically manipulate task difficulty during therapy. The search difficulty is progressively increased in 12 levels by modifying target/distractor similarity, with 4 levels at each of parallel (pop-out), complex and conjunction searches tasks. Examples of pop-out search include searching for either a T or an X amongst Os; or an H amongst Cs. Complex searches include searching for an S amongst Cs; an O amongst Gs; or a B amongst Ds. Both target shape and colour are altered in conjunction searches (searching for green X, amongst blue Xs and green Rs; or a green T amongst blue Ts and green upside-down Ts). In each trial, an example of target searched for is shown at the centre of the screen. Each level is also divided into 3 sub-levels, where set sizes are set to 8, 16 and 24 to increase task difficulty within each level. Each sub-level contains 200 trials, with target present or absent trials are presented in random order. Half of the trials are target present trials and speed and accuracy of target detection are recorded. The criterion for progression to the next level is achieving performance score of 80% in two of three sub-levels. If this performance criteria is not achieved the response time window is increased from 1500ms by 500ms when a level is repeated. Should a patient still not reach the required threshold, the limit on response time window is removed. Patients are encouraged to complete 3 sessions of training daily, each taking approximately 15 min.

### Pre- and post-therapy assessments

There are a number of tasks that are completed both before and after the therapy to assess the effect of the intervention. First, patients completed 10 practice trials. To obtain a baseline for visual exploration, 4 blocks of visual search (20 trials each) is presented at set sizes of 4, 8, 16 & 24. The median reaction time for each block is determined and averaged across those for the 4 blocks to obtain mean reaction time. The search error is calculated as the sum of errors (misreported present/absent) in all 4 blocks. A cancelation task was also used to obtain performance on a variation of the visual search task. In a cancelation task, patients are shown a scene containing 20 targets (e.g., diamonds) and 23 non-targets (e.g., 13 circles and 10 stars). The task is to click the mouse button on targets and press the space-bar when they believe they have marked all targets. This task is repeated 3 times. Ignoring the trials with longest and shortest times (i.e., the median of the three measurements), the time taken and number of errors made for the remaining trial is used as a measure of visual exploration.

It is beneficial to quantify the subjective effects of rehabilitation intervention in patients (Nelles et al. 2001). Using the same questionnaire as previously administered in similar studies (Nelles et al. 2001, 2009; Pambakian 2004; Sahraie et al. 2020) patients were asked to self-report their level of perceived difficulties in performing a range of activities of daily living on a 5-point scale, before and after therapy. The questions were on the level of difficulties in: difficulties seeing obstacles; bumping into obstacles; finding their way; finding objects on a table; finding objects in a room; finding objects in a supermarket; crossing the road; using public transport; reading or using a computer. The rating scale range was from no difficulty to having occasional, sometime, often or severe difficulties.

## Results

### Visual search reaction time

**Fig. 1:**
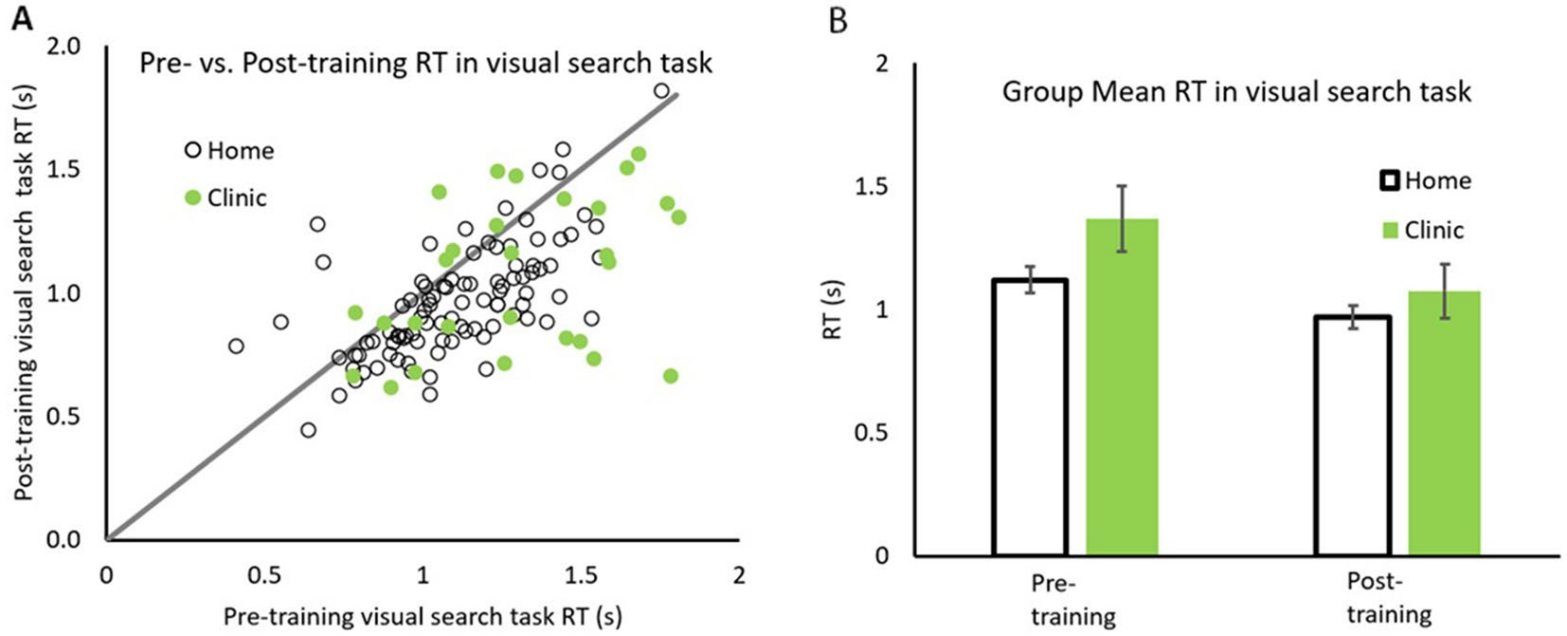
Comparison between pre/post training reaction times. Pre/post reaction times for both groups (**A**) and the group average RT (**B**) for home (open symbols) and in clinic (solid symbols) groups. Error bars represent 95% CI

Pre- vs. post-reaction times for each participant is plotted in Fig. 1A, with data for Home and Clinic based patients depicted in open and solid symbols respectively. The line with slope of unity representing equal performance before and after training. All patients whose data falls below the line showing faster reaction times after rehabilitation. The group mean of reaction times in visual search task are also plotted in Fig. 1B.

The majority of data points falling below the line indicate that 80/95 (84.2%) home and 23/30 (76.7%) clinic based patients had faster reaction times after than before rehabilitation. A repeated measure 2 × 2 ANOVA with factors patient groups (Home and Clinic) by Training (before and after) for Visual Search Reaction Time, revealed a significant main effect of training, *F*(1,123) = 67.86, *p* <.001, η_p_^2^ = 0.356. Home patients were significantly faster after rehabilitation (*M* = 0.971, *SD* = 0.235) than before (*M* = 1.121, *SD* = 0.262), (*M*_*diff*_=0.150, *SD* = 0.201), *t*(94) = 5.796, *p* <.001, Cohen’s d = 0.546. Similarly, Clinic patients were significantly faster after rehabilitation (*M* = 1.08, *SD* = 0.312) than before (*M* = 1.368, *SD* = 3.76), (*M*_*diff*_=0.285, *SD* = 0.374), *t*(29) = 6.192, *p* <.001, Cohen’s d = 1.038. Importantly there was a significant patient group x training interaction *F*(1,123) = 6.548, *p* =.012, η_p_^2^ = 0.051 indicating that clinic patients improved significantly more than Home based patients (Fig. 1B).

### Visual search error

**Fig. 2:**
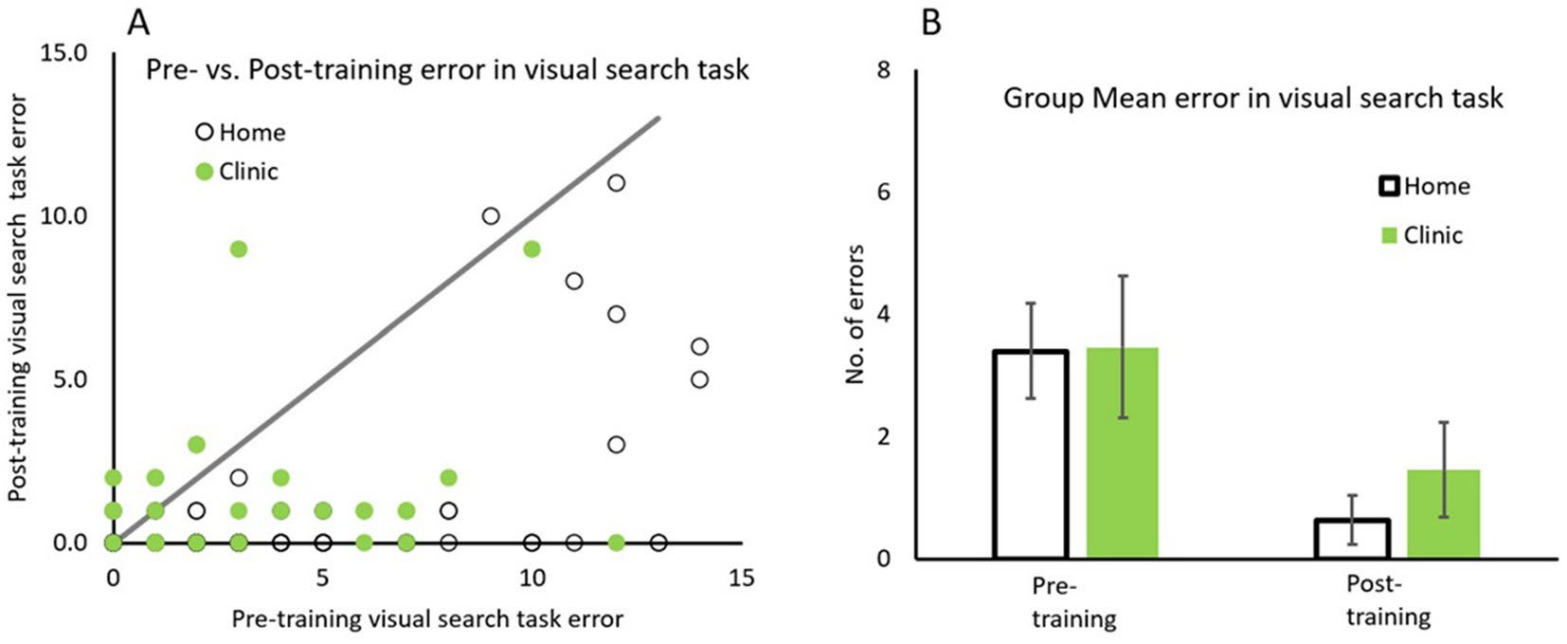
Comparison between pre/post training errors in visual search task. Pre/post errors in visual search task for both groups (**A**) and the group average error (**B**) for home (open symbols) and in clinic (solid symbols) groups. Error bars represent 95% CI

Pre- vs. post errors in visual search task for each participant is plotted in Fig. 2A, with data for Home and Clinic based patients depicted in open and solid symbols respectively. The line with slope of unity representing equal number of errors before and after training. All patients with data falling below the line have lower errors after rehabilitation. The group mean of errors in visual search task is also plotted in Fig. owin2B.

The majority of data points falling below the line indicates that 71/95 (74.7%) home and 19/30 (63.3%) clinic based patients had lower number of errors after than before rehabilitation. A repeated measure 2 × 2 ANOVA with factors patient groups (Home and Clinic) by Training (before and after) for Visual Search Error, revealed a significant main effect of training, *F*(1,123) = 48.15, *p* <.001, η_p_^2^ = 0.281). Home patients made significantly less error after rehabilitation (*M* = 0.63, *SD* = 2.01) than before (*M* = 3.40, *SD* = 3.85), (*M*_*diff*_=2.768, *SD* = 3.15), *t*(94) = 8.28, *p* <.001, Cohen’s d = 0.922. Similarly, Clinic patients had significantly less error after rehabilitation (*M* = 1.50, *SD* = 2.22) than before (*M* = 3.47, *SD* = 3.25), (*M*_*diff*_=1.97, *SD* = 0.65), *t*(29) = 3.306, *p* <.005, Cohen’s d = 0.655. There was no significant patient group x training interaction, *F*(1,123) = 1.380, *p* =.242, η_p_^2^ = 0.011. It is likely that this is due to overall lower number of errors made at post training. As shown in Fig. 2A, there is a spread of data along the horizontal axis, whereas the vertical distribution (post-training data) is much less spread.

### Cancelation task reaction time

**Fig. 3:**
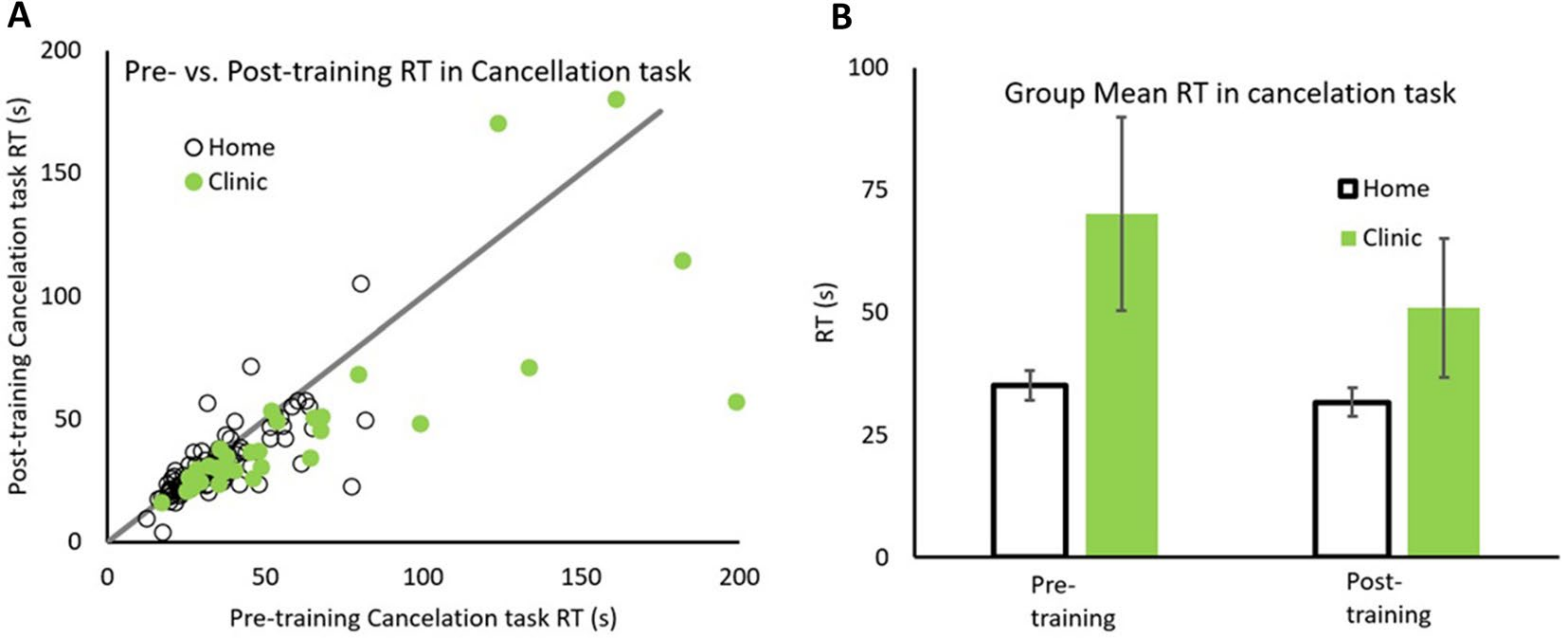
Comparison between pre/post training reaction time in Cancellation task. Distribution of pre/post reaction times (s) in the Cancellation task for both groups (**A**) and the group average reaction time (**B**) for home (open symbols) and in clinic (solid symbols) groups. Error bars represent 95%CI

Pre- vs. post-reaction times for cancelation task for each participant is plotted in Fig. 3A, with data for Home and Clinic based patients depicted in open and solid symbols respectively. The line with slope of unity representing equal performance before and after training. All patients with data falling below the line have faster reaction times while performing the cancelation task after rehabilitation. The group mean of reaction times in Cancelation Task are also plotted in Fig. 3B.

The majority of data points falling below the line indicate that 62/91 (68.1%) home and 25/31 (80.65%) clinic based patients had faster median reaction times in Cancelation Task after than before rehabilitation. A repeated measure 2 × 2 ANOVA with factors patient groups (Home and Clinic) by Training (before and after) for reaction time in Cancelation Task, revealed a significant main effect of training *F*(1,120) = 27.88, *p* <.001, η_p_^2^ = 0.189. Home patients were faster after rehabilitation (*M* = 31.70, *SD* = 14.28) than before (*M* = 35.13, *SD* = 14.98), but this difference was not significant (*M*_*diff*_=3.43, *SD* = 10.38), *t*(90) = 1.595, *p* =.679, Cohen’s d = 0.125. Similarly, Clinic patients were faster after rehabilitation (M = 50.99, SD = 40.29) than before (*M* = 70.07, *SD* = 55.87), and this difference was significant (*M*_*diff*_=19.08, *SD* = 36.85), *t*(30) = 5.182, *p* <.001, Cohen’s d = 0.695. Importantly similar to reaction time in the visual search task, there was a significant patient group x training interaction, *F*(1,120) = 13.478, *p* <.001, η_p_^2^ = 0.101, indicating that Clinic patients improved significantly more than the Home based patients (Fig. 3B).

### Disability score

**Fig. 4:**
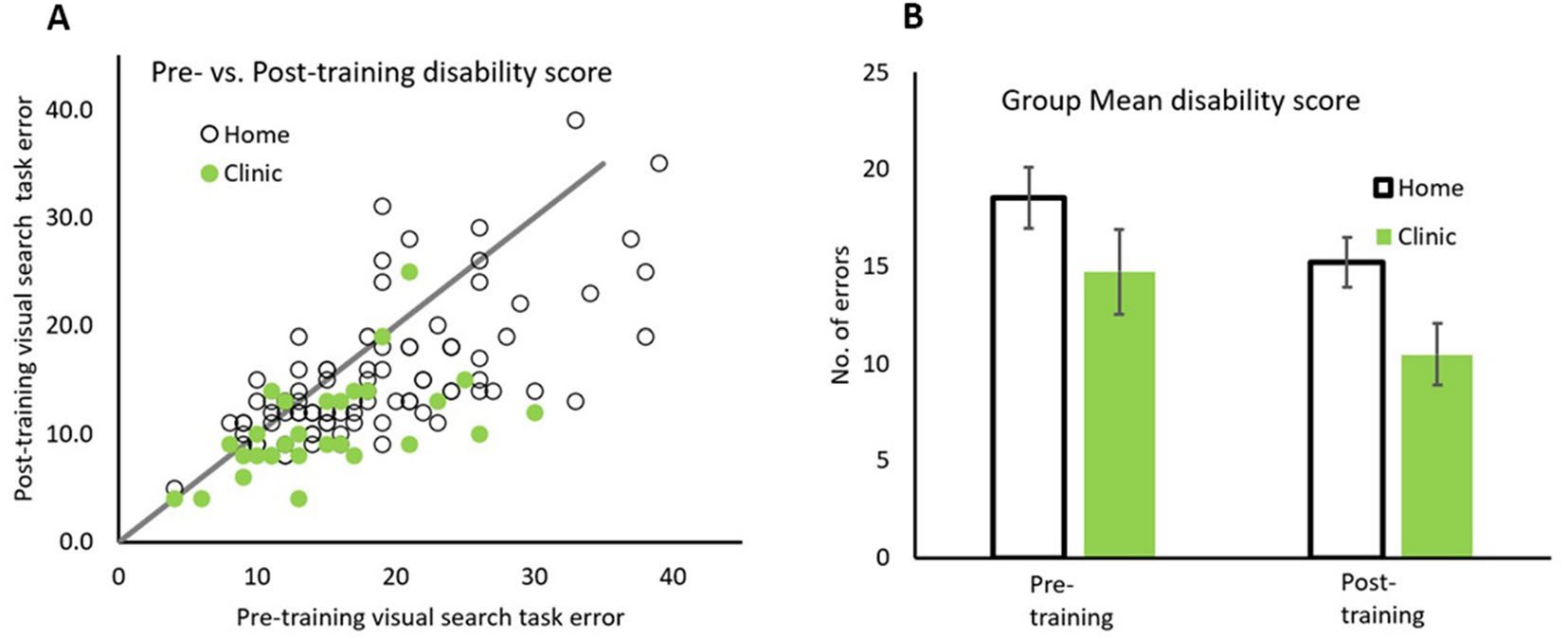
Comparison between pre/post training disability score. Pre/post disability scores for both groups (**A**) and the group average scores (**B**) for home (open symbols) and in clinic (solid symbols) groups. Error bars represent 95% CI

Pre- vs. post-Disability Score for each participant is plotted in Fig. 4A, with data for Home and Clinic based patients depicted in open and solid symbols respectively. The line with slope of unity representing equal levels of reported disability before and after training. All patients with data falling below the line had reported less disability after rehabilitation. The group mean of Disability Scores are also plotted in Fig. 4B.

The majority of data points falling below the line indicate that 60/90 (66.7%) home and 23/30 (76.7%) clinic setting patients reported less disability after than before rehabilitation. A repeated measure 2 × 2 ANOVA with factors patient groups (Home and Clinic) by Training (before and after) for Disability Score, revealed a significant main effect of training, *F*(1,118) = 41.74, *p* <.001, η_p_^2^ = 0.261. Home patients reported significantly lower Disability Score after rehabilitation (*M* = 15.21, *SD* = 6.19) than before (*M* = 18.54, *SD* = 7.56), (*M*_*diff*_=3.33, *SD* = 5.70), *t*(89) = 5.667, *p* <.001, Cohen’s d = 0.508. Similarly, Clinic patients reported significantly lower Disability Score after rehabilitation (M = 10.47, SD = 4.43) than before (M = 14.73, SD = 6.11), (M_*diff*_=4.267, SD = 5.18), *t*(29) = 4.188, *p* <.001, Cohen’s d = 0.650. However, there was no significant interaction of patient group x training, *F*(1,118) = 0.630, *p* =.429, η_p_^2^ = 0.005 (see Fig. 4B).

### Between group comparisons at baseline

Figures 1, 2, 3 and 4 above depict differences in baseline measurements between the two groups. Indeed independent sample comparisons of the home and clinic based patients show that the reaction times were significantly slower in the clinic group compared to the home group for both the cancelation task (t(120) = 5.45, *p* <.001) and in visual search task (t(120) = 4.03, *p* <.001), but the differences in errors made on both tasks were not significantly different. This may potentially indicate that the clinic group were more impaired compared to the home based group. However, the clinic group reported significantly lower subjective disability score compared to the home based group (t(118) = 2.499, *p* =.014). This dichotomy is discussed in the context of randomisation and differences between subjective and objectives measures of performance in the next section.

## Discussion

The ease at which healthy adults explore their environment with successive and seamless series of eye movements relies on the functioning of multiple mechanisms engaging in saccadic target detection, response preparation/movement planning and execution (Palmer et al. 2000; Rayner 1995). Sudden visual field loss, as a result of injury along the visual pathways, leads to disturbances of eye movements, even when the neuronal circuitry for response execution have remained intact (Hepworth et al. 2016). A similar set of disturbances can be observed under simulated visual field loss in healthy observers (Nowakowska et al. 2016, 2019). These combined observations point to high level origins of the mechanisms affected and are perhaps indicative of a lack of efficient strategy for interacting with the environment. The cornerstone of compensatory rehabilitation approaches is to aid the individual to learn new strategies that can lead to greater eye movement efficacy leading to faster exploration and interaction with the environment (Zihl 2010). The intervention reported here encourages patients to make large amplitude saccades into their field defect while searching the scene thereby adopting more efficient scene-exploration strategies. Combined manipulation of set-size, target-distractor similarity and response time window led to systematic changes in task difficulty, driving saccadic efficacy. In current study, NEC therapy was effective for the home group (*N* = 95) who showed faster reaction times in two search tasks, lower errors, and improved self-reported disability compared to pre-therapy, the extent of which were similar to improvements reported in an earlier study of 294 cases (Sahraie et al. 2020). The clinic group also showed improvement post therapy on the above measurements. Comparing the extent of improvements between groups, the objective measures (search times and cancelation task reaction time) showed larger improvements in those that underwent therapy in the clinic compared to those at home. Intriguingly their larger improvements measured objectively did not translate to perceived improvement in activities of daily living.

The outcomes of objective and subjective measures of sensitivity are of particular interest in the field of visual awareness (Kiefer and Kammer 2017; Kingdom and Prins 2016). In these circumstances, one is often interested in how the criterion free measures of objective detection tasks relate to subjective reports and experiences. The mapping of the performance across the measures are hotly debated (Schmidt and Biafora 2024), particularly as subjective criterion can play a part in many of seemingly objective tests of vision (Sahraie 2007). Another level of complexity is the relationship between subjective measures of detection, and perceived effects on the activity of daily living. For example, in relating changes of visual field (i.e., perimetry) to the effects on quality of life (McKean-Cowdin et al. 2007; van Gestel et al. 2010). The fundamental reason for using or promoting a compensatory rehabilitation strategy such as the one described here is the assumption that the use of such techniques could improve a patient’s interactions with their environment (Zihl 2010). Nevertheless, using subjective methods to determine the perceived effects are also problematic as they are open to experimenter effects (Rosenthal and Fode 1963), hence making it harder to relate to objective measures of performance. It remains likely that patients may rate their disability differently when reporting alone at home, compared to when interacting with clinicians in the clinical settings, leading to discrepancies between the objective and subjective measures reported at baseline in this study. For these reasons, there is a need for development and validation of functional assessment of visual function. Such assessments would be beneficial in future studies in quantifying changes in performance in daily tasks.

From a health delivery standpoint, the therapies delivered at the home environment are less costly, as they do not involve patient/carer transport and clinical staff time. The fundamental aspect of an online therapy is to ensure a low cost device that can be accessible to a wide range of patients. Therefore, at the design stage, it was fundamental to reduce the need for intervention by clinicians or technicians. Hence, the therapy relies on adherence of the patient to the instructions in all aspects of the therapy, from the initial setting of viewing distance and target sizes, to the subsequent correct usage. As a result, the parameters such as the actual visual angle of stimuli subtended could not be independently verifiable. This is a fundamental difference and limitation of low-cost tele-rehabilitation compared to clinic based interventions. Improving the accessibility and availability of treatments through at home options could also greatly benefit visually impaired patient populations, however further research is needed on this topic as limited evidence is available on the impact of telehealth and at-home treatment alternatives for visually impaired patient groups (Bittner et al. 2015). Conversely, there may be other benefits to the patients in undertaking a daily trip to the clinic, such as improved mental health by breaking the monotony of the daily routines and engaging socially with others, opportunity to raise concerns with the clinical team and overall enhanced motivation (Stronge et al. 2007). Indeed, we speculate that enhanced motivation may have played a critical role in attention which then resulted in better behavioural performance on the task (Schneider et al. 2018). However, based on a similar argument, one would expect larger reported subjective benefits also to follow in the clinic group. Some 67% of the home patients reported post-therapy subjective improvements compared to 77% of those who attended the clinic, nevertheless between group differences were not statistically significant. A limitation of the study was that neither the patients nor the authors could choose or allocate individuals to the groups. The lack of ability to randomise were basis for sample size differences and probably contributed to between group differences at the baseline.

A further imitation of the study is related to the determination of visual field loss. For the clinic group this was achieved by access to the medical records as the patients were already in the health system. In a drive to make the therapy accessible to the patient population, and in common with other compensatory therapies accessed online (Ong et al. 2015; Szalados et al. 2021) the home based patients were either referred by a medical practitioner after vision loss post brain injury, or had directly accessed the therapy online, hence the extent of the field defects were self-reported. With these caveats in mind, we conclude that NEC compensatory therapy is broadly effective in improving hemianopia patients’ outcomes, whether it is used in the home environment or in the clinical settings.

Finally, there are striking similarities between the performance of clinical samples reported previously (Sahraie et al. 2020) and those reported here. Although the majority of cases showed improvements (67–77%), there were several cases in both groups, in which the therapy had been ineffective. These are depicted on the scatter plots by symbols falling on or above the line with slope of one. Behavioural/psychological interventions often show efficacy in around 75% of participants with some 10% getting significantly worse following therapy (Amaral et al. 2022). The efficacy is often lower for many pharmaceutical interventions (Shetty et al. 2024). We have not been able to systematically study the reasons for deterioration in performance in our samples, there are however many possibilities ranging from general health deterioration, statistical artifacts due to variance, subsequent strokes, or other health complications. Overall, the findings demonstrate that patients with visual field loss can benefit from eye movement training, delivered remotely or under clinical supervision.

## Data Availability

Data for this study is deposited in Open Science Forum (osf.io/mpr4s) and will be made accessible to the public should the manuscript be accepted for publication.
